# Crystal structure, synthesis and thermal properties of bis­(aceto­nitrile-κ*N*)bis­(4-benzoyl­pyridine-κ*N*)bis­(iso­thio­cyanato-κ*N*)nickel(II)

**DOI:** 10.1107/S2056989019013756

**Published:** 2019-10-22

**Authors:** Carsten Wellm, Christian Näther

**Affiliations:** a Institut für Anorganische Chemie, Universität Kiel, Max-Eyth. Strasse 2, 241128 Kiel, Germany

**Keywords:** crystal structure, nickel(II) thio­cyanate, solvate, discrete com­plex, hydrogen bonding

## Abstract

In the crystal structure of the title com­pound, [Ni(NCS)_2_(CH_3_CN)_2_(C_12_H_9_NO)_2_] or Ni(NCS)_2_(4-benzoyl­pyridine)_2_(aceto­nitrile)_2_, the Ni^II^ ions are octa­hedrally coordinated by the N atoms of two thio­cyanate anions, two 4-benzoyl­pyridine ligands and two aceto­nitrile mol­ecules into discrete com­plexes that are located on centres of inversion. In the crystal, the discrete com­plexes are linked by centrosymmetric pairs of weak C—H⋯S hydrogen bonds into chains. Thermogravimetric measurements prove that, upon heating, the title com­plex loses the two aceto­nitrile ligands and transforms into a new crystalline modification of the chain com­pound [Ni(NCS)_2_(4-benzoyl­pyridine)_2_], which is different from that of the corresponding Co^II^, Ni^II^ and Cd^II^ coordination polymers reported in the literature. IR spectroscopic investigations indicate the presence of bridging thio­cyanate anions but the powder pattern cannot be indexed and, therefore, this structure is unknown.

## Abstract 2

In the crystal structure of the title com­pound, the Ni^II^ cations are octa­hedrally coordinated into discrete solvate com­plexes, that upon heating loses the aceto­nitrile ligands and transforms into an unknown modification of [Ni(NCS)_2_(4-benzoyl­pyridine)_2_].

## Chemical context   

**Figure 1:**
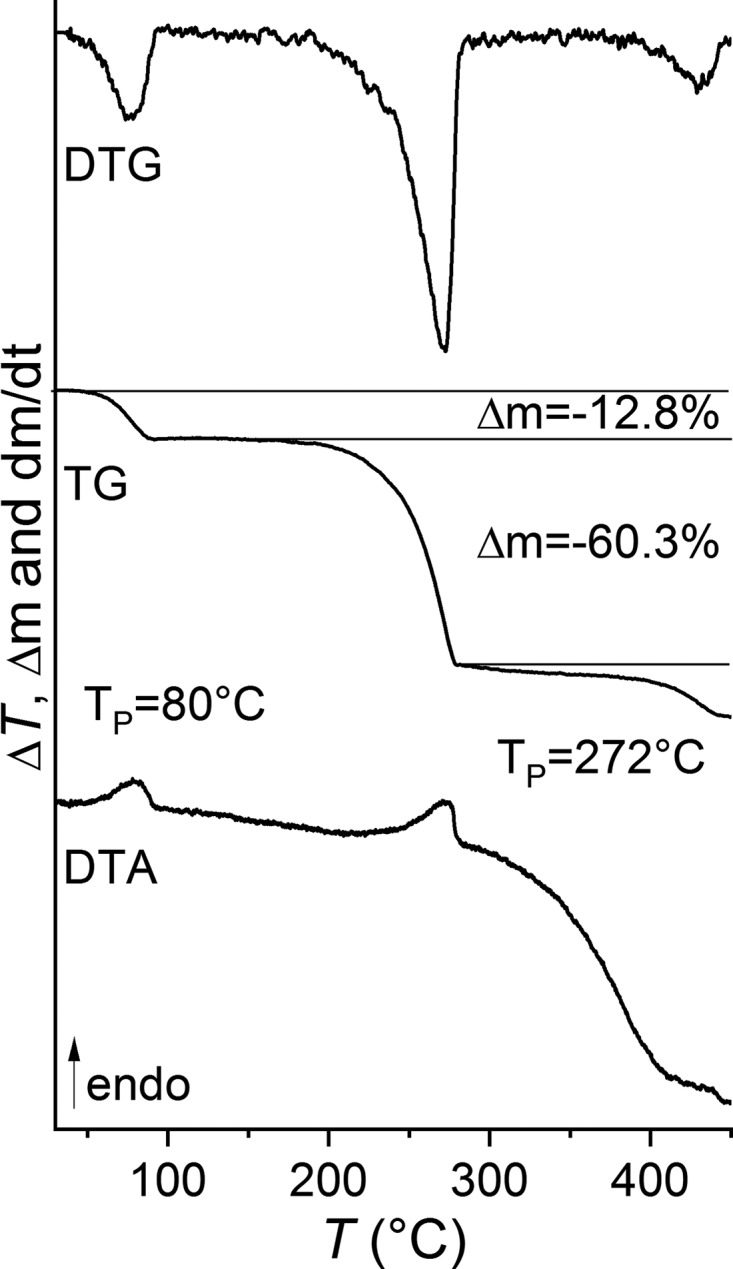
DTG, TG and DTA curve of the title com­pound with the experimental mass loss in % and the peak temperatures in °C. The calculated mass loss of two MeCN mol­ecules amounts to 13.2% and the loss of two 4-benzoyl­pyridine ligands corresponds to 58.8%.

In most cases, the synthesis of new coordination com­pounds is performed in solution, which in some cases leads to inhomogenous samples or some, *e.g.* metastable com­pounds, formed by kinetic control which can easily be overlooked. There are, however, some alternative routes, like synthesis *via* mol­ecular milling, molten flux synthesis, solid-gas reactions or thermal decom­position of suitable precursor com­pounds (Braga *et al.*, 2005 ▸, 2006 ▸; Näther *et al.*, 2013 ▸; Zurawski *et al.*, 2012 ▸; Höller *et al.*, 2008 ▸; Den *et al.*, 2019 ▸). These methods can have several advantages because, in most cases, they are irreversible, the products are obtained in qu­anti­tative yield, no solvent is needed and sometimes metastable isomeric or polymorphic modifications can be obtained. This is especially the case for thio­cyanate coordination polymers prepared by thermal decom­position of suitable precursor com­pounds that consist of com­plexes in which the anionic ligands are only terminally bonded and additionally coordinated by neutral N-donor co-ligands (Wöhlert *et al.*, 2014 ▸; Werner *et al.*, 2015 ▸). Upon heating, the co-ligands are stepwise removed, leading to new com­pounds in which the metal cations are linked by thio­cyanate anions into chains or layers (Neumann *et al.*, 2019 ▸). In this context, we have reported on coordination polymers based on 4-benzoyl­pyridine. In [*M*(NCS)_2_(4-benzoyl­pyri­dine)_2_] (*M* = Co and Ni) prepared in solution, a rare *cis*–*cis*–*trans* coordination is observed, in which the thio­cyanate N and S atoms are each in *cis* positions, whereas the co-ligand is *trans* (Rams *et al.*, 2017 ▸; Jochim *et al.*, 2018 ▸). This is in contrast to all other linear chain com­pounds, in which the coordinating atoms always show an *all*-trans coordination. Surprisingly, this coordination is found in [Cd(NCS)_2_(4-benzoyl­pyridine)_2_] (Neumann *et al.*, 2018 ▸). Therefore, the question arose if this form can be prepared with Ni by thermal decom­position using a suitable Ni^II^ precursor com­pound. One discrete com­plex with methanol has already been reported in the literature, but this com­pound cannot be prepared pure (Wellm & Näther, 2019*a* ▸). In the course of this project, we were able to prepare crystals from aceto­nitrile, which were characterized by single-crystal structure analysis, which proves that the title com­pound consists of discrete com­plexes with the com­position Ni(NCS)_2_(4-benzoyl­pyridine)_2_(aceto­nitrile)_2_. This com­pound can be prepared pure and is a promising precursor to prepare an Ni^II^ com­pound with bridging thio­cyanate anions (Fig. S1 in the supporting information). Measurements using differential thermoanalysis and thermogravimetry (DTA–TG) prove that on heating two mass steps are observed that are accom­panied by endothermic events in the DTA curve (Fig. 1 ▸). The experimental mass loss of 12.8% in the first step is in reasonable agreement with that calculated for the removal of two aceto­nitrile mol­ecules of 13.1%, indicating the formation of a com­pound with the desired com­position (Fig. 1 ▸). If the X-ray powder diffraction pattern of the residue formed after the first mass loss is com­pared with that calculated for [Ni(NCS)_2_(4-benzoyl­pyridine)_2_] reported in the literature, it is obvious that a crystalline phase has been formed (Fig. S1 in the supporting information). This new form is also different from [Cd(NCS)_2_(4-benzoyl­pyridine)_2_], indicating that a new isomeric or polymorphic form is obtained. The value of the CN stretching vibration of this form (2113 cm^−1^) is very different from that of the title com­pound (2080 cm^−1^) but com­parable to that observed in the known modification of [Ni(NCS)_2_(4-benzoyl­pyridine)_2_] (2121 cm^−1^) reported in the literature (Jochim *et al.*, 2018 ▸), which indicates a similar thio­cyanate coordination (Figs. S2, S3 and S4 in the supporting information). However, this powder pattern cannot be indexed and thus the structure of this new form is unknown.
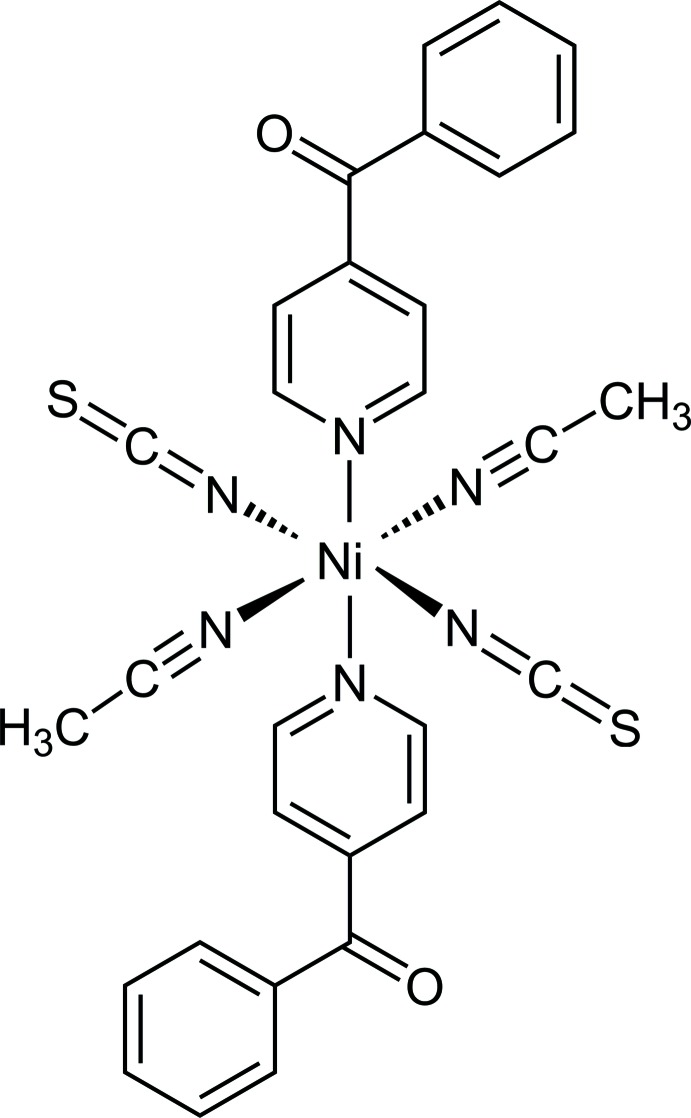


## Structural commentary   

**Figure 2:**
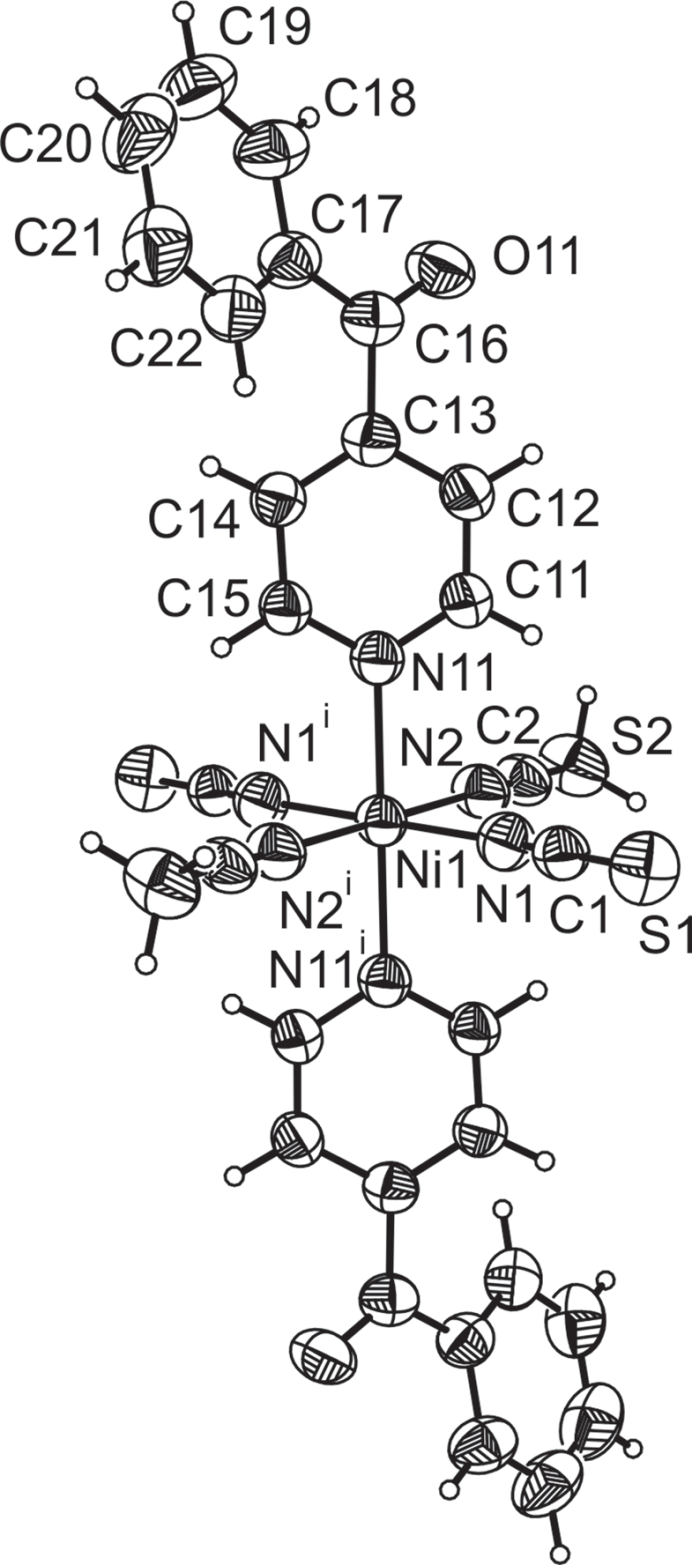
The mol­ecular structure of the title com­pound with labelling and displacement ellipsoids drawn at the 50% probability level. [Symmetry code: (i) −*x* + 2, −*y* + 1, −*z* + 2.]

The asymmetric unit of the title com­pound consists of one Ni^II^ ion that is located on a centre of inversion, as well as one thio­cyanate anion, one 4-benzoyl­pyridine co-ligand and one aceto­nitrile ligand that occupy general positions (Fig. 2 ▸). The Ni^II^ ions are sixfold coordinated by the N atoms of two terminal thio­cyanate anions, two 4-benzoyl­pyridine and two aceto­nitrile ligands (Fig. 2 ▸). The Ni—NCS bond length to the negatively charged anionic ligands of 2.038 (3) Å is shorter than the Ni—N(pyridine) and Ni—NCMe bond lengths of 2.108 (2) and 2.108 (2) Å, respectively (Table 1 ▸). The bond angles deviate only slightly from ideal values, which shows that the octa­hedra are only slightly distorted (Table 1 ▸). This is also obvious from the octa­hedral angle variance of 0.71 and the quadratic elongation of 1.0006 calculated according to a procedure published by Robinson *et al.* (1971 ▸). The dihedral angle between the carbonyl plane (C13/C16/C17/O11) and that of the phenyl (C17–C22) ring is 22.2 (2)°, and that between the planes of the pyridine ring (N11/C11–15) and the carbonyl group (C13/C16/C17/O11) is 33.7 (2)°, which shows that the 4-benzoyl­pyridine ligand is not coplanar.

## Supra­molecular features   

**Figure 3:**
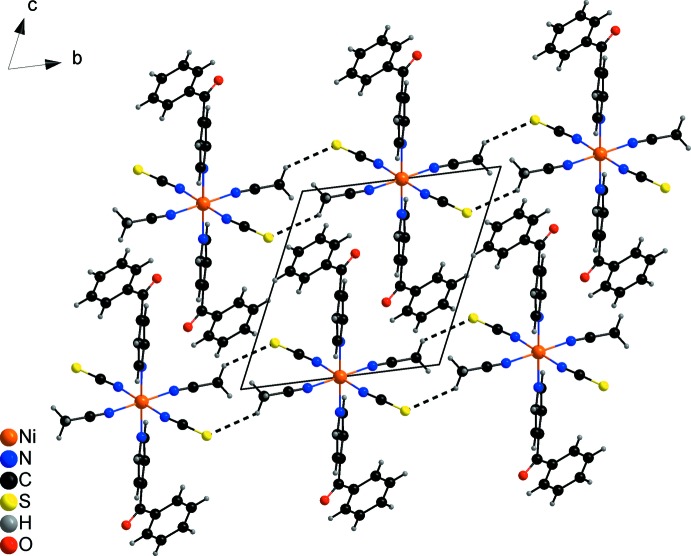
Part of the crystal structure of the title com­pound, viewed along the crystallographic *a* axis, and with inter­molecular C—H⋯S hydrogen bonding shown as dashed lines.

The discrete com­plexes are arranged into columns that proceed along the crystallographic *a* axis (Fig. 3 ▸). Along the *b* axis they are linked into chains by centrosymmetric pairs of weak C—H⋯S hydrogen bonds between the aceto­nitrile H atoms and the thio­cyanate S atoms (Fig. 3 ▸ and Table 2 ▸).

## Database survey   

There are already some com­pounds reported in the Cam­bridge Structural Database (Groom *et al.*, 2016 ▸) that consist of transition-metal thio­cyanates and 4-benzoyl­pyridine ligands. These are Zn(NCS)_2_(4-benzoyl­pyridine)_2_ with tetra­hedrally coordinated Zn^II^ cations (Neumann *et al.*, 2018 ▸) and Cu(NCS)_2_(4-benzoyl­pyridine)_2_ in which the Cu^II^ cations are square-planar coordinated (Bai *et al.*, 2011 ▸). There are also a number of discrete com­plexes with an octa­hedral metal coordination and terminal thio­cyanate anions (Drew *et al.*, 1985 ▸; Soliman *et al.*, 2014 ▸; Wellm & Näther, 2018 ▸, 2019*a* ▸,*b* ▸; Neumann *et al.*, 2018 ▸; Suckert *et al.*, 2017 ▸). Finally, there are several coordination polymers with the com­position [*M*(NCS)_2_(4-benzoyl­pyridine)_2_]_*n*_ (*M* = Cd^II^, Ni^II^ and Co^II^), in which the cations are linked by pairs of μ-1,3-coordinating thio­cyanate anions into chains (Neumann *et al.*, 2018 ▸; Rams *et al.*, 2017 ▸; Jochim *et al.*, 2018 ▸).

## Synthesis and crystallization   

Ba(SCN)_2_·3H_2_O and 4-benzoylpyridine were purchased from Alfa Aesar. Ni(SO_4_)·6H_2_O was purchased from Merck. All solvents and reactants were used without further purification.

Ni(NCS)_2_ was prepared by the reaction of equimolar amounts of Ni(SO_4_)·6H_2_O and Ba(SCN)_2_·3H_2_O in water. The resulting white precipitate of BaSO_4_ was filtered off, and the solvent was evaporated from the filtrate. The green solid was dried at room temperature.

### Synthesis   

Crystals of the title com­pound suitable for single-crystal X-ray diffraction were obtained by the reaction of Ni(NCS)_2_ (26.2 mg, 0.15 mmol) with 4-benzoyl­pyridine (27.5 mg, 0.15 mmol) in aceto­nitrile (1.5 ml) for 2 d at 354 K in a closed test tube. A polycrystalline powder was obtained by stirring a solution of Ni(NCS)_2_ (87.4 mg, 0.5 mmol) and 4-benzoyl­pyridine (183.2 mg, 1.0 mmol) in MeCN (3 ml) for 4 d.

### Experimental details   

Differential thermoanalysis and thermogravimetry (DTA–TG) were performed under a dynamic nitro­gen atmosphere in Al_2_O_3_ crucibles using an STA PT1600 thermobalance from Linseis. The XRPD measurements were performed using a Stoe Transmission Powder Diffraction System (STADI P) with Cu *K*α radiation that was equipped with a linear position-sensitive MYTHEN detector from Stoe & Cie. The IR data were measured using a Bruker Alpha-P ATR–IR spectrometer.

## Refinement   

The C—H hydrogens were positioned with idealized geometry (methyl H atoms allowed to rotate but not to tip) and refined with *U*_iso_(H) = 1.2*U*_eq_(C) (1.5 for methyl H atoms) using a riding model. Crystal data, data collection and structure refinement details are summarized in Table 3 ▸.

## Supplementary Material

Crystal structure: contains datablock(s) I. DOI: 10.1107/S2056989019013756/lh5928sup1.cif

Structure factors: contains datablock(s) I. DOI: 10.1107/S2056989019013756/lh5928Isup2.hkl

Additional figures. DOI: 10.1107/S2056989019013756/lh5928sup3.pdf

CCDC references: 1958279, 1958279

Additional supporting information:  crystallographic information; 3D view; checkCIF report

## Figures and Tables

**Table 1:** Selected geometric parameters (Å, °)

Ni1—N1^i^	2.038 (3)	Ni1—N2^i^	2.093 (2)
Ni1—N1	2.038 (3)	Ni1—N11^i^	2.108 (2)
Ni1—N2	2.093 (2)	Ni1—N11	2.108 (2)
			
N1^i^—Ni1—N1	180.0	N1^i^—Ni1—N11	89.97 (9)
N1^i^—Ni1—N2	91.36 (9)	N1—Ni1—N11	90.03 (9)
N1—Ni1—N2	88.64 (9)	N2—Ni1—N11	89.69 (8)
N1^i^—Ni1—N2^i^	88.64 (9)	N2^i^—Ni1—N11	90.31 (8)
N1—Ni1—N2^i^	91.36 (9)	N11^i^—Ni1—N11	180.0
N2—Ni1—N2^i^	180.0	C1—N1—Ni1	163.8 (2)
N1^i^—Ni1—N11^i^	90.03 (9)	C15—N11—Ni1	121.05 (18)
N1—Ni1—N11^i^	89.97 (9)	C11—N11—Ni1	121.64 (17)
N2—Ni1—N11^i^	90.31 (8)	C2—N2—Ni1	171.5 (2)
N2^i^—Ni1—N11^i^	89.69 (8)		

Symmetry code: (i) 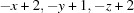.

**Table 2:** Hydrogen-bond geometry (Å, °)

*D*—H⋯*A*	*D*—H	H⋯*A*	*D*⋯*A*	*D*—H⋯*A*
C3—H3*B*⋯S1^ii^	0.98	2.98	3.662 (3)	127

Symmetry code: (ii) 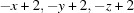.

**Table 3:** Experimental details

Crystal data	
Chemical formula	[Ni(NCS)_2_(C_2_H_2_N_21_)_2_(C_12_H_9_NO)_2_]
*M*_r_	623.38
Crystal system, space group	Triclinic, *P* 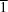
Temperature (K)	200
*a*, *b*, *c* (Å)	7.2716 (5), 10.4868 (6), 10.8677 (6)
α, β, γ (°)	65.540 (4), 88.893 (5), 88.378 (5)
*V* (Å^3^)	754.02 (8)
*Z*	1
Radiation type	Mo *K*α
μ (mm^−1^)	0.82
Crystal size (mm)	0.14 × 0.05 × 0.04

Data collection	
Diffractometer	Stoe IPDS2
Absorption correction	Numerical (*X-SHAPE* and *X-RED32*; Stoe & Cie, 2008 ▸)
*T*_min_, *T*_max_	0.837, 0.966
No. of measured, independent and observed [*I* > 2σ(*I*)] reflections	9692, 3283, 2634
*R*_int_	0.041
(sin θ/λ)_max_ (Å^−1^)	0.639

Refinement	
*R*[*F* ^2^ > 2σ(*F* ^2^)], *wR*(*F* ^2^), *S*	0.046, 0.100, 1.06
No. of reflections	3283
No. of parameters	188
H-atom treatment	H-atom parameters constrained
Δρ_max_, Δρ_min_ (e Å^−3^)	0.28, −0.40

Computer programs: *X-AREA* (Stoe & Cie, 2008 ▸), *SHELXS97* (Sheldrick, 2008 ▸), *SHELXL2014* (Sheldrick, 2015 ▸), *XP* in *SHELXTL* (Sheldrick, 2008 ▸) and *DIAMOND* (Brandenburg, 1999 ▸) and *publCIF* (Westrip, 2010 ▸).

## supplementary crystallographic information

### Crystal data

**Table:**

[Ni(NCS)_2_(C_2_H_2_N_21_)_2_(C_12_H_9_NO)_2_]	*Z* = 1
*M**_r_* = 623.38	*F*(000) = 322
Triclinic, *P*1	*D*_x_ = 1.373 Mg m^−^^3^
*a* = 7.2716 (5) Å	Mo *K*α radiation, λ = 0.71073 Å
*b* = 10.4868 (6) Å	Cell parameters from 9692 reflections
*c* = 10.8677 (6) Å	θ = 2.1–25.2°
α = 65.540 (4)°	µ = 0.82 mm^−^^1^
β = 88.893 (5)°	*T* = 200 K
γ = 88.378 (5)°	Needle, blue
*V* = 754.02 (8) Å^3^	0.14 × 0.05 × 0.04 mm

### Data collection

**Table:**

Stoe IPDS-2 diffractometer	2634 reflections with *I* > 2σ(*I*)
ω scans	*R*_int_ = 0.041
Absorption correction: numerical (X-SHAPE and X-RED32; Stoe & Cie, 2008)	θ_max_ = 27.0°, θ_min_ = 2.1°
*T*_min_ = 0.837, *T*_max_ = 0.966	*h* = −9→9
9692 measured reflections	*k* = −13→13
3283 independent reflections	*l* = −13→13

### Refinement

**Table:**

Refinement on *F*^2^	0 restraints
Least-squares matrix: full	Hydrogen site location: inferred from neighbouring sites
*R*[*F*^2^ > 2σ(*F*^2^)] = 0.046	H-atom parameters constrained
*wR*(*F*^2^) = 0.100	*w* = 1/[σ^2^(*F*_o_^2^) + (0.0383*P*)^2^ + 0.2958*P*] where *P* = (*F*_o_^2^ + 2*F*_c_^2^)/3
*S* = 1.06	(Δ/σ)_max_ = 0.001
3283 reflections	Δρ_max_ = 0.28 e Å^−^^3^
188 parameters	Δρ_min_ = −0.40 e Å^−^^3^

### Special details

**Table:**

Geometry. All esds (except the esd in the dihedral angle between two l.s. planes) are estimated using the full covariance matrix. The cell esds are taken into account individually in the estimation of esds in distances, angles and torsion angles; correlations between esds in cell parameters are only used when they are defined by crystal symmetry. An approximate (isotropic) treatment of cell esds is used for estimating esds involving l.s. planes.

### Fractional atomic coordinates and isotropic or equivalent isotropic displacement parameters (Å^2^)

**Table:**

	*x*	*y*	*z*	*U*_iso_*/*U*_eq_	
Ni1	1.0000	0.5000	1.0000	0.04329 (15)	
N1	1.1925 (3)	0.6472 (3)	0.9052 (2)	0.0544 (5)	
C1	1.2695 (4)	0.7524 (3)	0.8535 (3)	0.0476 (6)	
S1	1.38122 (12)	0.89701 (8)	0.77890 (9)	0.0675 (2)	
N11	0.8623 (3)	0.5571 (2)	0.8151 (2)	0.0470 (5)	
C11	0.9542 (4)	0.5796 (3)	0.6999 (3)	0.0511 (6)	
H11	1.0844	0.5686	0.7029	0.061*	
C12	0.8685 (4)	0.6177 (3)	0.5778 (3)	0.0516 (6)	
H12	0.9387	0.6322	0.4987	0.062*	
C13	0.6780 (4)	0.6350 (3)	0.5711 (3)	0.0476 (6)	
C14	0.5829 (4)	0.6119 (3)	0.6894 (3)	0.0501 (6)	
H14	0.4527	0.6230	0.6889	0.060*	
C15	0.6789 (3)	0.5726 (3)	0.8082 (3)	0.0476 (6)	
H15	0.6116	0.5558	0.8891	0.057*	
C16	0.5863 (4)	0.6676 (3)	0.4381 (3)	0.0538 (6)	
C17	0.4134 (4)	0.7550 (3)	0.4013 (3)	0.0553 (7)	
C18	0.3648 (4)	0.8489 (3)	0.4564 (3)	0.0615 (7)	
H18	0.4388	0.8554	0.5246	0.074*	
C19	0.2080 (5)	0.9336 (4)	0.4122 (4)	0.0777 (10)	
H19	0.1755	0.9989	0.4492	0.093*	
C20	0.0997 (5)	0.9225 (4)	0.3144 (4)	0.0886 (12)	
H20	−0.0074	0.9806	0.2840	0.106*	
C21	0.1459 (5)	0.8281 (4)	0.2610 (4)	0.0868 (12)	
H21	0.0694	0.8199	0.1949	0.104*	
C22	0.3025 (5)	0.7452 (3)	0.3024 (3)	0.0700 (8)	
H22	0.3350	0.6814	0.2637	0.084*	
O11	0.6554 (3)	0.6210 (2)	0.3620 (2)	0.0687 (6)	
N2	0.8453 (3)	0.6523 (2)	1.0367 (2)	0.0540 (6)	
C2	0.7727 (4)	0.7466 (3)	1.0432 (3)	0.0518 (6)	
C3	0.6820 (5)	0.8675 (3)	1.0510 (3)	0.0715 (9)	
H3A	0.7191	0.9524	0.9733	0.107*	
H3B	0.7171	0.8740	1.1348	0.107*	
H3C	0.5484	0.8581	1.0501	0.107*	

### Atomic displacement parameters (Å^2^)

**Table:**

	*U*^11^	*U*^22^	*U*^33^	*U*^12^	*U*^13^	*U*^23^
Ni1	0.0442 (3)	0.0443 (3)	0.0446 (3)	0.00743 (19)	−0.00183 (19)	−0.0221 (2)
N1	0.0536 (13)	0.0556 (13)	0.0542 (14)	0.0029 (11)	−0.0014 (11)	−0.0230 (11)
C1	0.0488 (14)	0.0509 (15)	0.0481 (15)	0.0079 (12)	−0.0038 (12)	−0.0259 (12)
S1	0.0725 (5)	0.0514 (4)	0.0826 (6)	−0.0061 (4)	0.0032 (4)	−0.0317 (4)
N11	0.0465 (11)	0.0490 (12)	0.0479 (12)	0.0070 (9)	−0.0016 (9)	−0.0230 (10)
C11	0.0458 (13)	0.0620 (16)	0.0476 (15)	0.0031 (12)	0.0021 (11)	−0.0249 (13)
C12	0.0515 (14)	0.0575 (15)	0.0472 (15)	0.0018 (12)	0.0033 (12)	−0.0233 (12)
C13	0.0523 (14)	0.0456 (13)	0.0457 (14)	0.0028 (11)	−0.0034 (11)	−0.0199 (11)
C14	0.0475 (13)	0.0547 (15)	0.0508 (15)	0.0052 (11)	−0.0033 (12)	−0.0249 (12)
C15	0.0453 (13)	0.0539 (14)	0.0457 (14)	0.0072 (11)	−0.0006 (11)	−0.0234 (12)
C16	0.0590 (16)	0.0534 (15)	0.0479 (15)	−0.0010 (12)	−0.0051 (13)	−0.0197 (12)
C17	0.0565 (15)	0.0528 (15)	0.0472 (15)	−0.0032 (12)	−0.0049 (12)	−0.0110 (12)
C18	0.0587 (17)	0.0571 (16)	0.0586 (18)	0.0031 (13)	−0.0003 (14)	−0.0141 (14)
C19	0.069 (2)	0.066 (2)	0.080 (2)	0.0120 (16)	0.0040 (18)	−0.0139 (17)
C20	0.061 (2)	0.082 (2)	0.089 (3)	0.0105 (18)	−0.0111 (19)	−0.001 (2)
C21	0.068 (2)	0.089 (3)	0.076 (2)	−0.0072 (19)	−0.0239 (19)	−0.006 (2)
C22	0.074 (2)	0.0656 (19)	0.0601 (19)	−0.0066 (16)	−0.0159 (16)	−0.0149 (15)
O11	0.0799 (14)	0.0802 (14)	0.0541 (12)	0.0092 (11)	−0.0061 (11)	−0.0363 (11)
N2	0.0574 (13)	0.0568 (13)	0.0527 (13)	0.0123 (11)	−0.0055 (11)	−0.0282 (11)
C2	0.0606 (16)	0.0522 (15)	0.0468 (15)	0.0120 (13)	−0.0042 (12)	−0.0252 (12)
C3	0.093 (2)	0.0588 (17)	0.067 (2)	0.0288 (17)	−0.0073 (17)	−0.0328 (15)

### Geometric parameters (Å, º)

**Table:**

Ni1—N1^i^	2.038 (3)	C16—O11	1.217 (3)
Ni1—N1	2.038 (3)	C16—C17	1.494 (4)
Ni1—N2	2.093 (2)	C17—C18	1.383 (4)
Ni1—N2^i^	2.093 (2)	C17—C22	1.395 (4)
Ni1—N11^i^	2.108 (2)	C18—C19	1.390 (4)
Ni1—N11	2.108 (2)	C18—H18	0.9500
N1—C1	1.164 (3)	C19—C20	1.379 (5)
C1—S1	1.626 (3)	C19—H19	0.9500
N11—C15	1.339 (3)	C20—C21	1.371 (6)
N11—C11	1.343 (3)	C20—H20	0.9500
C11—C12	1.373 (4)	C21—C22	1.376 (5)
C11—H11	0.9500	C21—H21	0.9500
C12—C13	1.391 (4)	C22—H22	0.9500
C12—H12	0.9500	N2—C2	1.135 (3)
C13—C14	1.381 (4)	C2—C3	1.445 (4)
C13—C16	1.505 (4)	C3—H3A	0.9800
C14—C15	1.380 (4)	C3—H3B	0.9800
C14—H14	0.9500	C3—H3C	0.9800
C15—H15	0.9500		
			
N1^i^—Ni1—N1	180.0	N11—C15—C14	123.2 (2)
N1^i^—Ni1—N2	91.36 (9)	N11—C15—H15	118.4
N1—Ni1—N2	88.64 (9)	C14—C15—H15	118.4
N1^i^—Ni1—N2^i^	88.64 (9)	O11—C16—C17	121.0 (3)
N1—Ni1—N2^i^	91.36 (9)	O11—C16—C13	118.7 (2)
N2—Ni1—N2^i^	180.0	C17—C16—C13	120.3 (2)
N1^i^—Ni1—N11^i^	90.03 (9)	C18—C17—C22	119.4 (3)
N1—Ni1—N11^i^	89.97 (9)	C18—C17—C16	122.7 (3)
N2—Ni1—N11^i^	90.31 (8)	C22—C17—C16	117.9 (3)
N2^i^—Ni1—N11^i^	89.69 (8)	C17—C18—C19	120.1 (3)
N1^i^—Ni1—N11	89.97 (9)	C17—C18—H18	120.0
N1—Ni1—N11	90.03 (9)	C19—C18—H18	120.0
N2—Ni1—N11	89.69 (8)	C20—C19—C18	119.8 (4)
N2^i^—Ni1—N11	90.31 (8)	C20—C19—H19	120.1
N11^i^—Ni1—N11	180.0	C18—C19—H19	120.1
C1—N1—Ni1	163.8 (2)	C21—C20—C19	120.3 (3)
N1—C1—S1	178.2 (2)	C21—C20—H20	119.9
C15—N11—C11	117.3 (2)	C19—C20—H20	119.9
C15—N11—Ni1	121.05 (18)	C20—C21—C22	120.5 (4)
C11—N11—Ni1	121.64 (17)	C20—C21—H21	119.8
N11—C11—C12	123.0 (2)	C22—C21—H21	119.8
N11—C11—H11	118.5	C21—C22—C17	120.0 (4)
C12—C11—H11	118.5	C21—C22—H22	120.0
C11—C12—C13	119.4 (3)	C17—C22—H22	120.0
C11—C12—H12	120.3	C2—N2—Ni1	171.5 (2)
C13—C12—H12	120.3	N2—C2—C3	179.4 (4)
C14—C13—C12	117.8 (2)	C2—C3—H3A	109.5
C14—C13—C16	123.6 (2)	C2—C3—H3B	109.5
C12—C13—C16	118.4 (2)	H3A—C3—H3B	109.5
C15—C14—C13	119.3 (2)	C2—C3—H3C	109.5
C15—C14—H14	120.4	H3A—C3—H3C	109.5
C13—C14—H14	120.4	H3B—C3—H3C	109.5

Symmetry code: (i) −*x*+2, −*y*+1, −*z*+2.

### Hydrogen-bond geometry (Å, º)

**Table:**

*D*—H···*A*	*D*—H	H···*A*	*D*···*A*	*D*—H···*A*
C3—H3*B*···S1^ii^	0.98	2.98	3.662 (3)	127

Symmetry code: (ii) −*x*+2, −*y*+2, −*z*+2.
