# Supplementary material for: Crystal structure, synthesis and thermal properties of bis­(aceto­nitrile-κN)bis­(4-benzoyl­pyridine-κN)bis­(iso­thio­cyanato-κN)nickel(II)
Source: Acta Crystallogr E Crystallogr Commun. 2019 Oct 22;75(Pt 11):1685–8. doi: 10.1107/S2056989019013756 (PMC6829719; doi:10.1107/S2056989019013756)

STRUCTURE REPORTS

Volume 75 (2019)

Supporting information for article:

Crystal structure, synthesis and thermal properties of bis(acetonitrile-κ*N*)bis(4-benzoylpyridine-κ*N*)bis(isothiocyanato-κ*N*)nickel(II)

Carsten Wellm and Christian Näther

Figure S1. Calculated (A) and experimental (B) powder pattern of the title compound, as well as the calculated powder patterns for $[Ni(NCS)_2$ (4-benzoylpyridine$)_2]_n$ (C) and $[Cd(NCS)_2$(4-benzoylpyridine$)_2]_n$ (D) together with the experimental powder pattern of the residue obtained after the first mass loss in a TG measurementof the title compound (E).

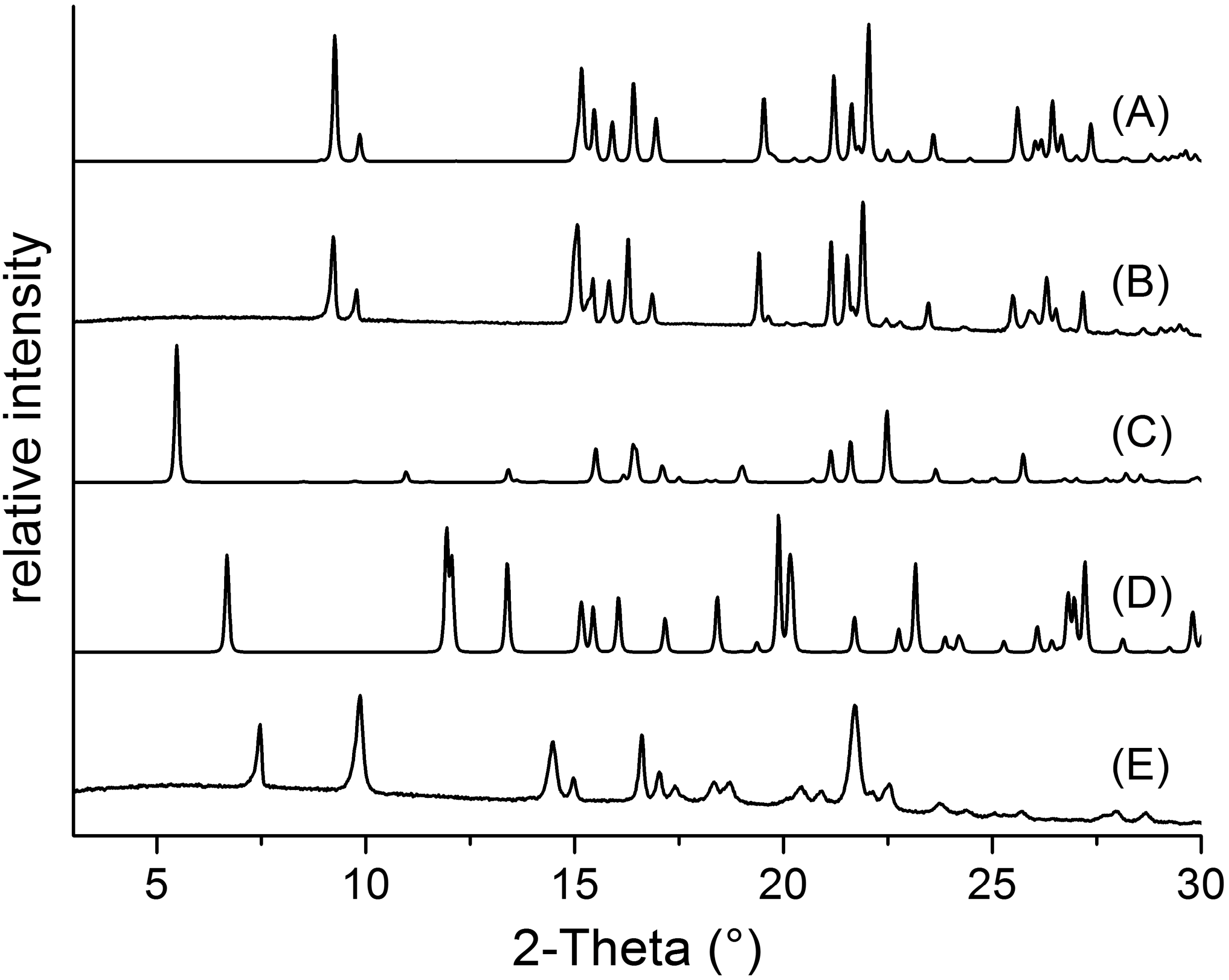

Figure S2. IR spectrum of the title compound.

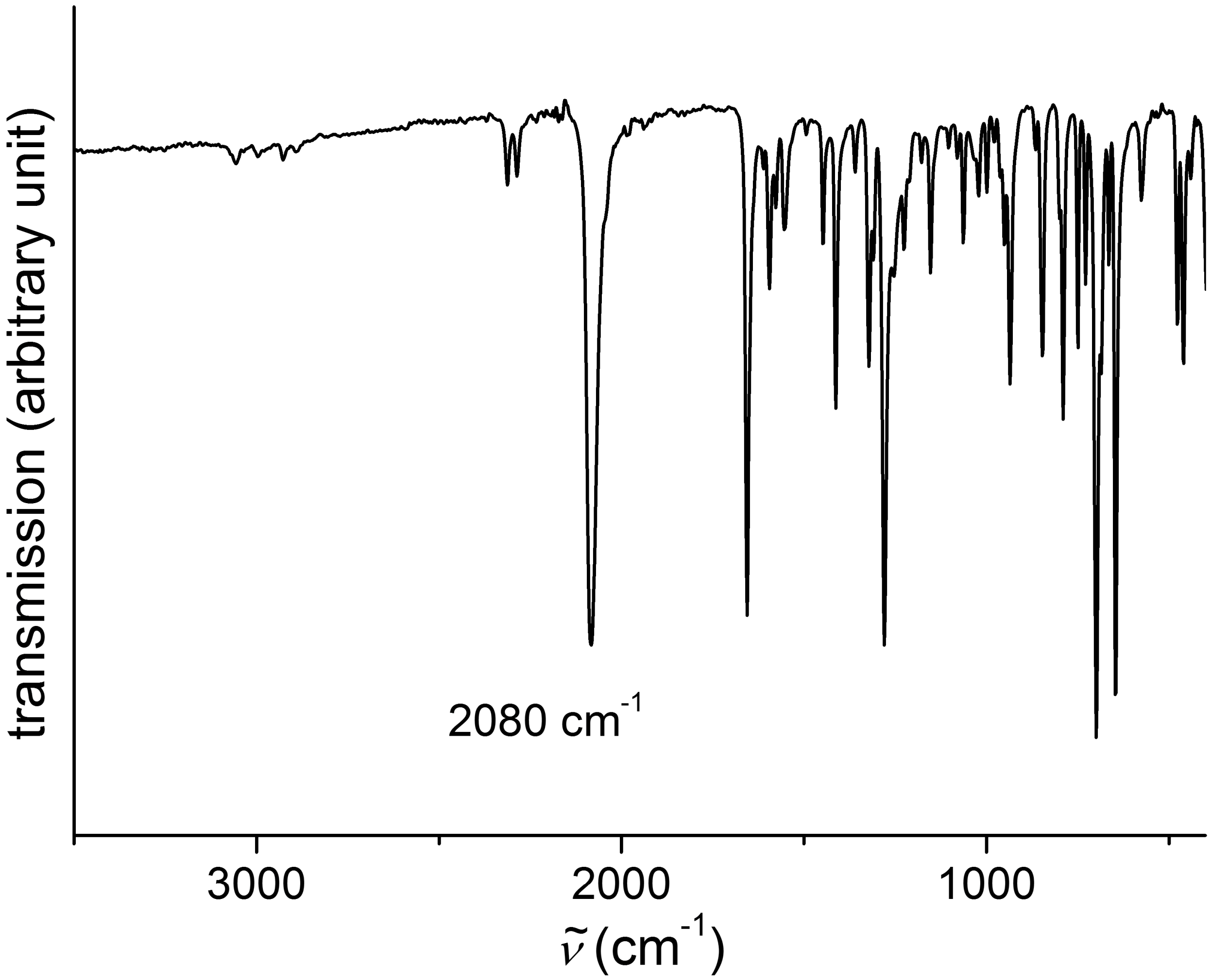

Figure S3. IR spectrum of the residue obtained by thermogravimetry.

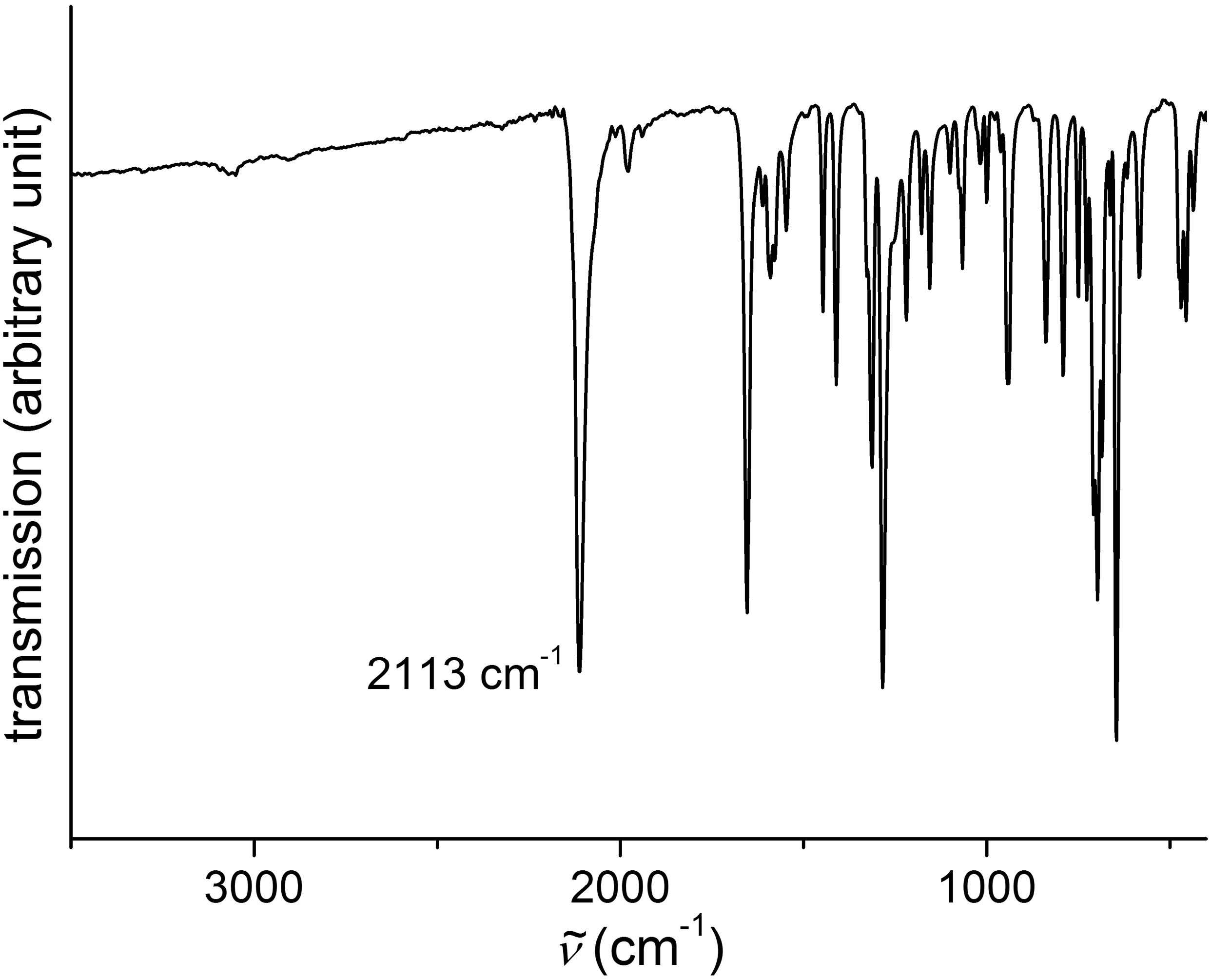

Figure S4. IR spectrum of [Ni(NCS)_2 (4-benzoylpyridine)_2]n reported in the literature.

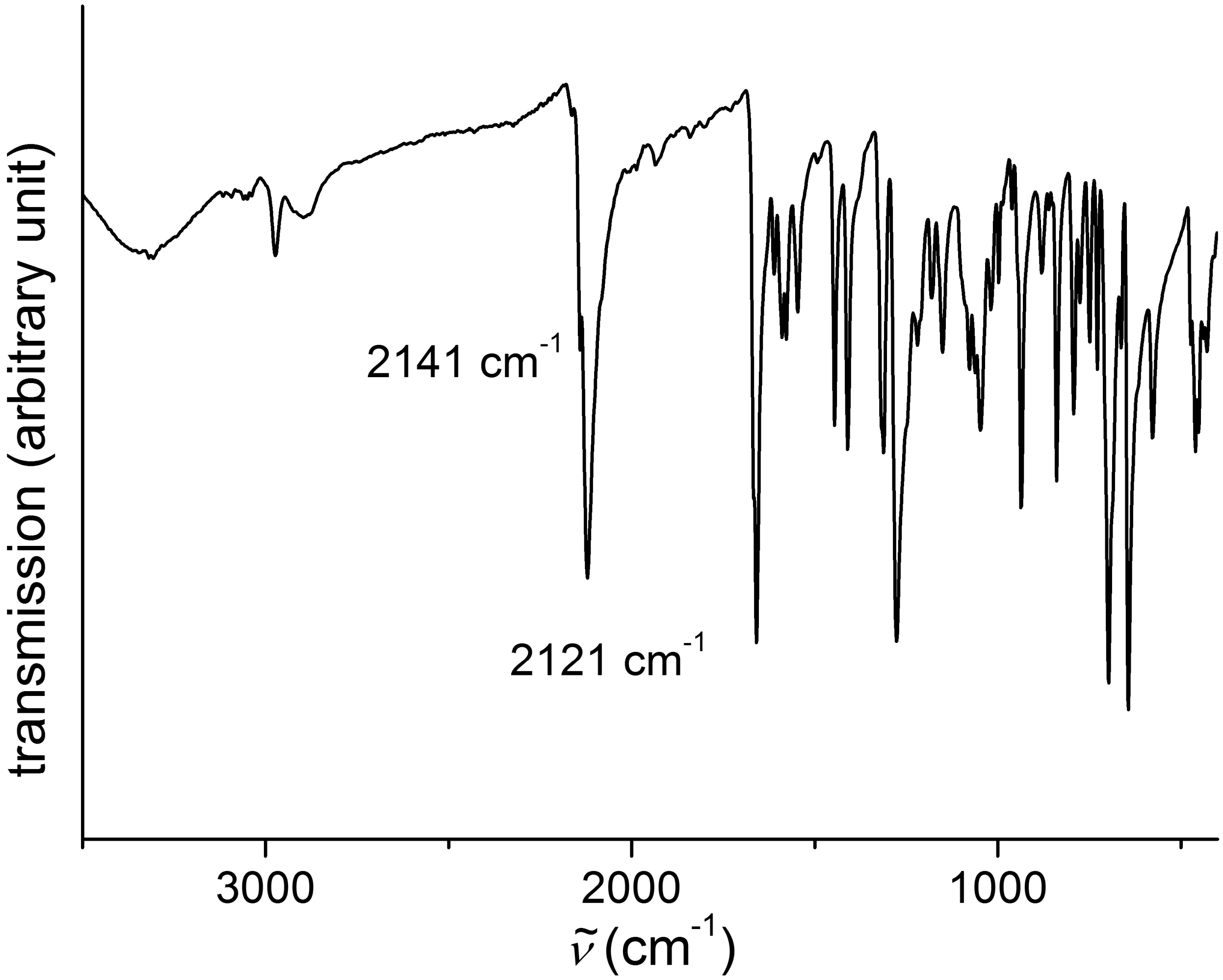

Supplement: Supplementary file 3 [file e-75-01685-sup3.pdf]
